# Non-interfacial self-assembly of synthetic protocells

**DOI:** 10.1186/s40824-023-00402-w

**Published:** 2023-07-03

**Authors:** Xiaolin Xu, Wencai Guan, Xiaolei Yu, Guoxiong Xu, Chenglong Wang

**Affiliations:** 1 Research Center for Clinical Medicine, Jinshan Hospital, Fudan University, Shanghai, 201508 P.R. China; 2 The State Key Laboratory of Metal Matrix Composites, School of Materials Science and Engineering, Shanghai Jiao Tong University, Shanghai, 200240 P. R. China

**Keywords:** Non-interfacial self-assembly, Protocell, mRNA vaccine, Cancer immunotherapy, Calcium homeostasis

## Abstract

**Background:**

Protocell refers to the basic unit of life and synthetic molecular assembly with cell structure and function. The protocells have great applications in the field of biomedical technology. Simulating the morphology and function of cells is the key to the preparation of protocells. However, some organic solvents used in the preparation process of protocells would damage the function of the bioactive substance. Perfluorocarbon, which has no toxic effect on bioactive substances, is an ideal solvent for protocell preparation. However, perfluorocarbon cannot be emulsified with water because of its inertia.

**Methods:**

Spheroids can be formed in nature even without emulsification, since liquid can reshape the morphology of the solid phase through the scouring action, even if there is no stable interface between the two phases. Inspired by the formation of natural spheroids such as pebbles, we developed non-interfacial self-assembly (NISA) of microdroplets as a step toward synthetic protocells, in which the inert perfluorocarbon was utilized to reshape the hydrogel through the scouring action.

**Results:**

The synthetic protocells were successfully obtained by using NISA-based protocell techniques, with the morphology very similar to native cells. Then we simulated the cell transcription process in the synthetic protocell and used the protocell as an mRNA carrier to transfect 293T cells. The results showed that protocells delivered mRNAs, and successfully expressed proteins in 293T cells. Further, we used the NISA method to fabricate an artificial cell by extracting and reassembling the membrane, proteins, and genomes of ovarian cancer cells. The results showed that the recombination of tumor cells was successfully achieved with similar morphology as tumor cells. In addition, the synthetic protocell prepared by the NISA method was used to reverse cancer chemoresistance by restoring cellular calcium homeostasis, which verified the application value of the synthetic protocell as a drug carrier.

**Conclusion:**

This synthetic protocell fabricated by the NISA method simulates the occurrence and development process of primitive life, which has great potential application value in mRNA vaccine, cancer immunotherapy, and drug delivery.

**Graphical Abstract:**

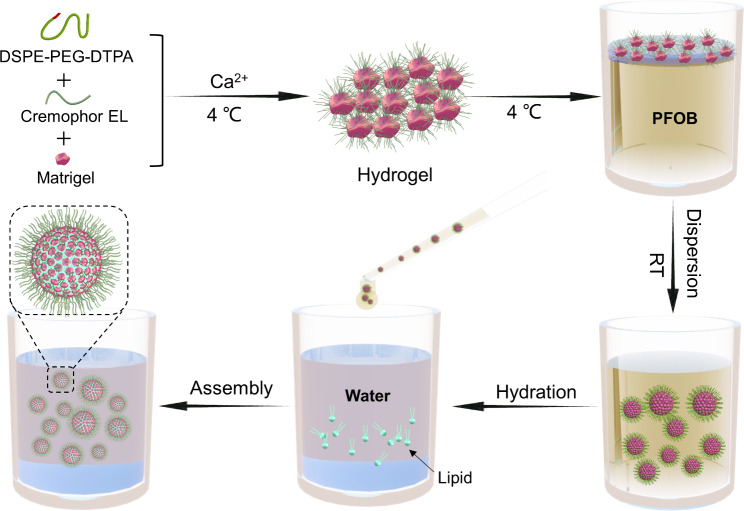

**Supplementary Information:**

The online version contains supplementary material available at 10.1186/s40824-023-00402-w.

## Introduction

Protocell refers to the basic unit of life with cell structure and function and also refers to “synthetic molecular assembly with cell structure and function” [1–3]. Protocells can be derived from lipids, polymers, lipid/polymer mixtures, colloidosome, metal-organic frame materials, and natural cell membranes [4]. Protocell construction methods can be divided into top-down and bottom-up methods. The top-down approach uses biological methods to remove genes and organelles of a living cell or to replace the original genome with a synthetic one to obtain a protocell. The bottom-up approach is to build protocells from scratch, by chemically assembling organic biomolecules or inorganic materials to build microvesicles with cellular mimicry [5]. Although these two approaches are different, they complement each other. Synthetic protocells can be constructed and used as biomimetic models for cell-free gene expression, artificial cytoskeletal remodeling, membrane gating, motility, enzyme activity, and membrane growth and division [6]. In general, the membrane assembly of protocells is realized by the spontaneous or oriented organization of various supramolecular and nano-scale components with a continuous semi-permeable shell and a closed water-filled interior [7]. Researchers have constructed protocell microcapsules with membrane-like structures by using fatty acid, protein, inorganic nanoparticles, synthetic polymer, and other components [8–10], and used them in the synthesis, regulation, and transport of biomolecules [8, 11, 12]. Thus, protocell as a new kind of biological functional material shows great application potential.

The protocells have great applications in the field of mRNA vaccines. mRNA vaccine is a new vaccine technology actively explored all over the world [13, 14]. mRNA has no infection risks, which can be degraded by normal cells, thus improving vaccination safety [15]. In addition, the mRNA vaccine is produced through an in vitro cell-free transcription process [16, 17], so it can more effectively respond to the demand for vaccine products in the case of an outbreak [18–20]. Furthermore, mRNA vaccines work like a drug production machine to guide cells to produce specific proteins [21–24], which exert the system’s efficacy and improve the immune effect [25]. Because of the unique cavities, the protocells can be used as a vessel for mRNA vaccine production and delivery.

Cellular immunotherapy is a method that separates immune cells from a donor body to fight against cancer cells or viruses in the patient’s body [26–28]. It also refers to the method that extracts the antigen cells such as cancer cells, and injects them back into the patient’s body after cultured and modified in vitro, to enhance the immunoreaction [29, 30]. However, current autologous cancer cell-based vaccines show poor immunogenicity [31]. Cancer vaccine produced by protocell technology greatly preserves the immunogenicity of cancer antigens in the case of inactivation of cancer cells, so as to enhance the ability of the patient’s self immune system to recognize and kill cancer cells.

Furthermore, protocells could deliver genes or drugs to treat difficult-to-cure diseases [32]. However, some organic solvents used in the preparation process of protocells will damage the function of the bioactive substance. Perfluorocarbon, which has no toxic effects on bioactive substances, is an ideal solvent for protocell preparation, but it cannot be emulsified with water because of its inertia. Since liquid can reshape the morphology of the solid phase through the scouring action, even if there is no stable interface between the two phases. Inspired by the formation of pebbles in nature, we developed the non-interfacial self-assembly (NISA) of microdroplets as a step toward synthetic protocells. Unlike interface self-assembly, which is carried out at the interface of two phases, non-interfacial self-assembly refers to the process by which molecules or particles spontaneously organize into an ordered structure independent of the interface between two phases. Non-interface self-assembly method has the advantages of great flexibility and easy control, which is widely used in the field of nanotechnology to prepare various nanostructures, such as nanoparticles, nanotubes, nanowires, nanocrystals, and nanosheets. 1,2-Distearoyl-sn-glycerol-3-phosphoethanolamine-Poly(ethylene glycol)-Diethylenetriamine penlaacetic acid (DSPE-PEG-DTPA), Cremophor EL and Matrigel were selected to form the protocells. DSPE-PEG-DTPA and Cremophor EL are amphiphilic compounds with hydrophilic groups of polyethylene glycol (PEG) and polyethylene oxide (PEO). Studies have shown that carriers modified with PEG can significantly extend the cycle time and improved the stability in vivo [33]. Matrigel is liquid at 4 ℃ but gradually turns into a gel above 10 ℃, which is conducive to the preparation of protocells and maintaining the stability of the protocells.

## Results

### Fabrication of synthetic protocells upon NISA method

**Fig. 1:**
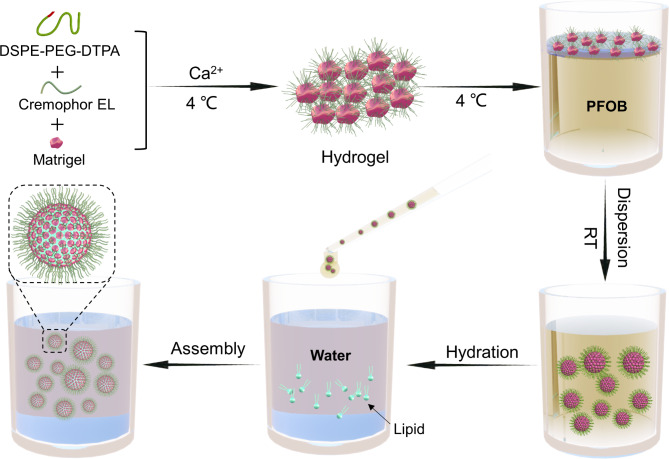
Schematic illustration depicting the fabrication of NISA-based synthetic protocells

We developed a NISA-based synthetic protocells preparation technique, as shown in Fig. 1, specifically, DSPE-PEG-DTPA (the structure shown in Fig. S1), Cremophor EL, and Matrigel were mixed. Then the mixed hydrogel was chelated with calcium ions at 4 ℃ and the hydrogel was immediately transferred into perfluorooctyl bromide (PFOB), followed by shaking at room temperature to solidify the hydrogel (Fig. S2). The hydrogel was then dispersed in an aqueous solution containing lipid molecules. Finally, the protocells were formed after the lipid was wrapped onto the hydrogel.

**Fig. 2:**
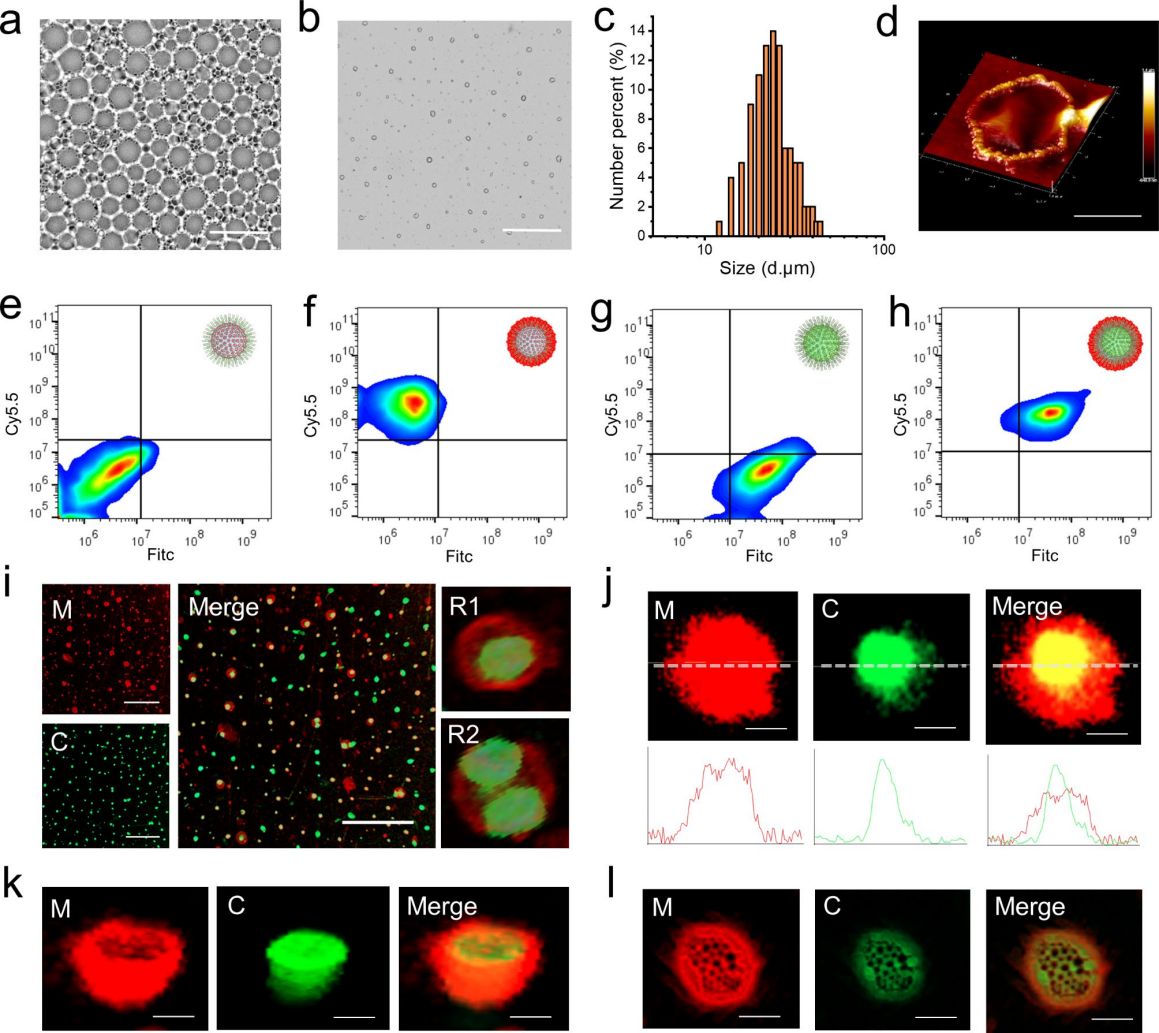
Characterization of synthetic protocells. **(a)** Microscopic photograph of hydrogel dispersing in PFOB. **(b)** Microscopic photograph of the synthetic protocells. **(c)** The particle size distribution of the synthetic protocells. **(d)** Atomic force microscopy image of the collapsed synthetic protocell. Scale bar, 10 μm. **e-h.** Flow cytometry data for synthetic protocells with no fluorescence labeling **(e)**, Cy5.5 labeled lipids **(f)**, FITC labeled hydrogel **(g)**, Cy5.5 labeled lipids and FITC labeled hydrogel **(h)**. **i.** Confocal images of synthetic protocells. M reflects the membrane, C reflects the core, R1 and R2 are representative synthetic protocells. Scale bar, 100 μm. **j.** Confocal images of a synthetic protocell. Graphs at the bottom show corresponding line profiles of fluorescence intensities across the synthetic protocell. Scale bar, 10 μm. **k.** 3D confocal fluorescence reconstructions of a synthetic protocell. Scale bar, 10 μm. **l.** 3D confocal fluorescence reconstructions of a dried synthetic protocell. Scale bar, 10 μm

**Fig. 3:**
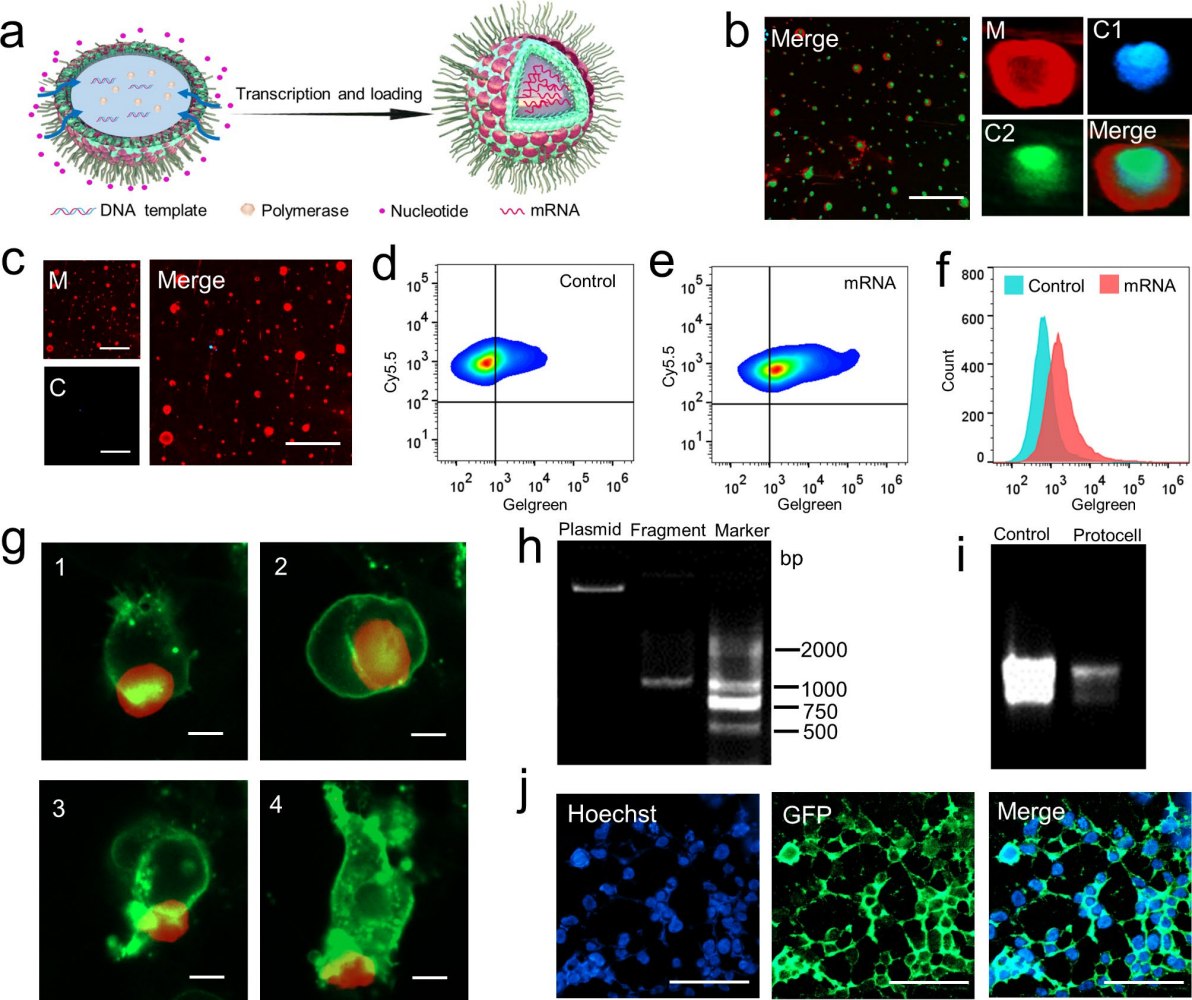
mRNA transcription in synthetic protocells and expression in native cells. **(a)** Schematic illustration of mRNA transcription in synthetic protocells. **(b)** Confocal images of synthetic protocell post-transcription. M reflects the membrane, C1 and C2 reflect the core in the blue and green channels respectively. Scale bar, 100 μm. **(c)** Confocal images of synthetic protocell post-transcription, with no nucleotide addition during the transcription. Scale bar, 100 μm. **d-f.** Flow cytometry data for synthetic protocells post-transcription **(d)**, no nucleotides addition group as control **(e)**, histogram reflects the difference in mRNA transcription **(f)**. **g.** Dynamic changes of 293T cells incubating with synthetic protocells in 4 h. Red reflects synthetic protocells, green reflects 293T cells, and 1, 2, 3, 4 are dynamic in order. Scale bar, 10 μm. **h.** Agarose gel electrophoresis of GFP fragment amplified from the plasmid. **i.** Agarose gel electrophoresis of GFP-mRNA transcribed in synthetic protocells, compared with GFP-mRNA transcribed by transcription kit. **j.** Confocal images of 293T cells incubated with GFP-mRNA loaded synthetic protocell for 24 h. Scale bar, 100 μm

Through microscope observation, we found that the gel microspheres were dispersed in the spaces between PFOB droplets (Fig. 2a) because PFOB could not emulsify the hydrogel. Then the mixture was transferred into an aqueous solution containing lipid molecules of dioleoylphosphatidylethanolamine (DOPE) and 1,2-dioleoyl-3-trimethyl-ammonium-propane (DOTAP), synthetic protocells were formed after the gel microspheres encapsulated by lipid molecules (Fig. 2b), with particle sizes ranging from 10 to 50 microns (Fig. 2c). The dehydrated synthetic protocell was observed with collapsed cystic structures by atomic force microscopy (Fig. 2d). We further labeled the synthetic protocells using a fluorescent dye, the lipid molecules on the surface were labeled by Cy5.5, and the internal gel was labeled with FITC. Flow cytometry detection proved that the synthetic protocells were successfully prepared (Fig. 2e h). Then confocal laser observation was carried out, and the images showed that the synthetic protocells have a very similar structure to native cell, a red lipid membrane wrapped around the green gel (Fig. 2i, Video S1), and the synthetic protocells also mimics the structure of a dividing cell (R2 in Fig. 2i). The cell-like structure was further verified by fluorescence intensities distribution across the synthetic protocell (Fig. 2j) and 3D confocal fluorescence reconstructions (Fig. 2k). In addition, we found that the synthetic protocells exhibited internal pore structure after drying (Fig. 2l, Video S2), which was the result of internal gel dehydration. All of the above results demonstrate that the synthetic protocells were successfully obtained by using NISA-based protocell techniques, with the morphology very similar to native cells.

### mRNA transcription in synthetic protocells

In order to solve the problems of easy degradation of mRNA and low load efficiency in the mRNA carrier, an approach of protocell-based mRNA bomb was explored by using the NISA method to fabricate synthetic protocells. As shown in Fig. 3a, because the membrane of the synthetic protocell is semi-permeable, ribonucleotides can pass through the membrane to transcribe mRNA with the help of a DNA template and RNA transcriptase inside the protocell. Confocal laser images showed that mRNA was successfully transcribed in protocells, which also proved that DNA template and RNA transcriptase had been successfully encased in the protocell. Since the emission wavelength of DAPI combined with mRNA was around 500 nm, we successfully viewed mRNA in blue and green channels (Fig. 3b, Video S3). There was no fluorescence in the control protocells without nucleotide addition (Fig. 3c). The flow cytometry results also proved that the mRNA was successfully expressed when nucleotides were added (Fig. 3d and f). In order to verify the potential of the synthetic protocells as mRNA transporter, we incubated the synthetic protocells with 293T cells and observed the dynamic process of the interaction between the protocells and 293T cells. The video shows protocell attempts to enter the cell (Fig. 3g, Video S4), by binding to the surface of the cell enabling the protocell easy to carry material into the cell. We then amplified a sequence expressing green fluorescent protein (GPF) by PCR as a DNA template (Fig. 3h), after digesting the protocells to extract the mRNA, gel electrophoresis was carried out. The result showed that mRNA was successfully transcribed within 4 h, although the product of mRNA transcribed in protocells was less (3.9 ng/µL) than that of the in vitro transcription kit (35.8 ng/µL), the band was narrower, indicating that less mRNA debris is generated, proving that the protocells had a protective effect on the mRNA (Fig. 3i). In order to verify whether the protocells could transport mRNA into native cells and express proteins, we incubated the protocells with 293T cells for 24 h, and the results showed that GFP was successfully expressed (Fig. 3j). Therefore, the results indicated that NISA-based technique achieved mRNA transcription and loading in synthetic protocells, and successfully expressed proteins in native cells as an mRNA carrier.

### Fabrication of artificial cancer cells via the NISA method

**Fig. 4:**
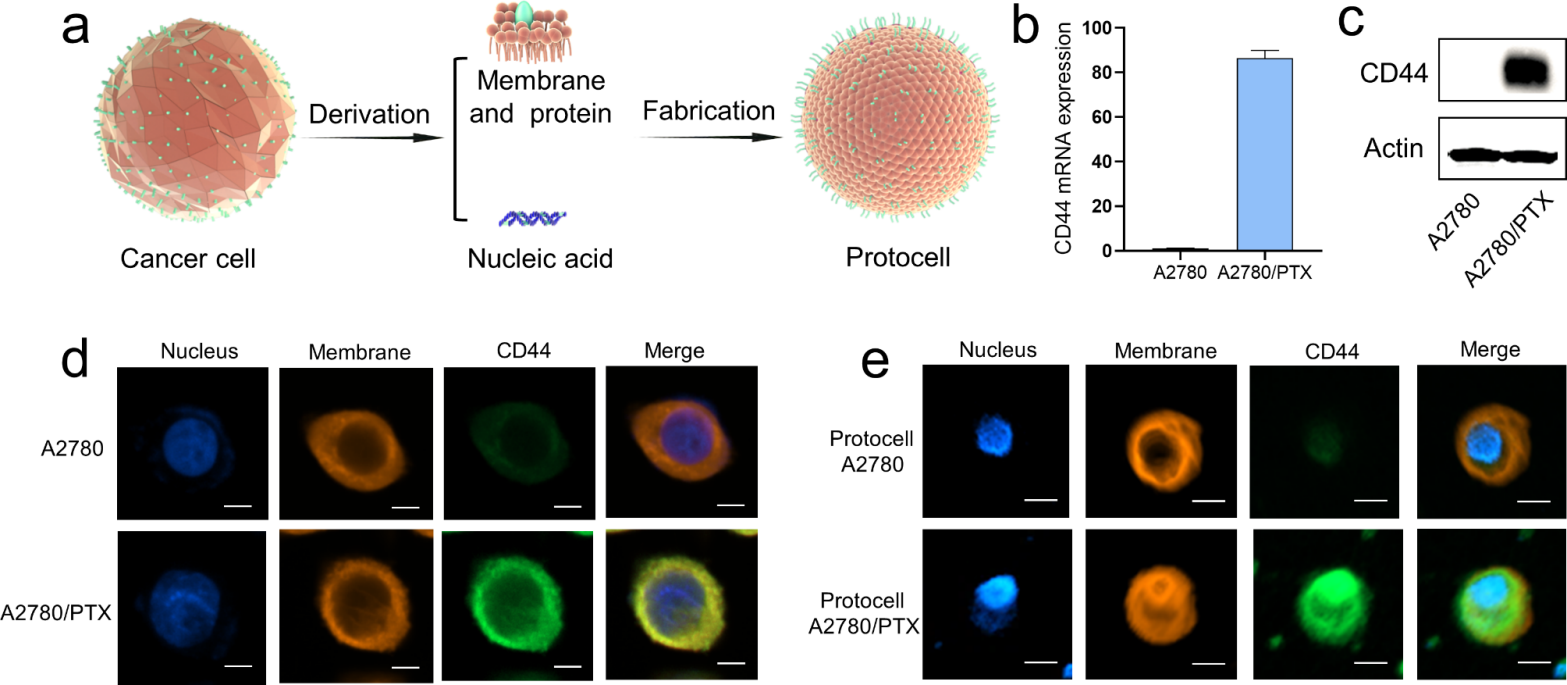
Fabrication of artificial cancer cells via NISA method. **(a)** Schematic illustration of artificial cancer cell fabricated by NISA method. **(b)** The expression of CD44 mRNA was higher in A2780/PTX compared with its sensitive counterpart A2780 detected by qRT-PCR. **(c)** The expression of CD44 protein was higher in A2780/PTX compared with A2780 determined by Western blot. **(d)** Laser confocal micrograph of A2780 and A2780/PTX, CD44 protein was highly expressed in A2780/PTX analyzed by immunofluorescence. Scale bar, 5 μm. **(e)** Laser confocal micrograph of protocell A2780 and protocell A2780/PTX and the expression of CD44 protein was analyzed by immunofluorescence. Scale bar, 5 μm

Although synthetic protocells can simulate the morphology of cells, many functional structural units of cells, such as transmembrane proteins, glycoproteins, and lipid valves, are difficult to be simulated. Therefore, in order to obtain these functional units and make the protocells more similar to real cells, we use the NISA method to build artificial cells. As tumors often develop resistance during treatment, drug-resistant tumors are difficult to be cured [34]. It is important for immunotherapy to lysate tumor cells and then inject them back into the body for immunity [35]. To maximize the antigenicity of tumor cells, we tried to reconstruct tumor cells via the NISA-based protocell method, the schematic of the fabrication process is shown in Fig. 4a. CD44 is a membrane protein that is highly expressed in drug-resistant ovarian cancer cells [36]. It was proved that CD44 was highly expressed in paclitaxel-resistant A2780 cells (A2780/PTX) by qRT-PCR (Fig. 4b) and western blot (Fig. 4c). After lysis of A2780 and A2780/PTX cells, the cell membrane and total DNA were extracted respectively, and then NISA-based protocell technology was utilized for recombination of the tumor cells. Cell membranes were stained with membrane fluorescent pigment, and the nucleus was stained with Hoest33342. An immunofluorescence assay was performed using FITC-labeled anti-CD44. The results showed that the tumor protocell was with a similar structure as that of the native tumor cell (Fig. 4d and e, Figs. S3-S4, Videos S5-S6), with DNA located in the core and the membrane wrapped on the surface, and the distribution of CD44 in protocell was also consistent with that of native tumor cell. Artificial cancer cells can maintain their morphological stability within three months stored at 4 ℃, while collapse significantly stored at 4 ℃ for a year (Fig. S5). The results indicated that the recombination of tumor cells was successfully achieved by using NISA-based protocell technology.

### Restoring calcium homeostasis and reversing chemoresistance by synthetic protocells

**Fig. 5:**
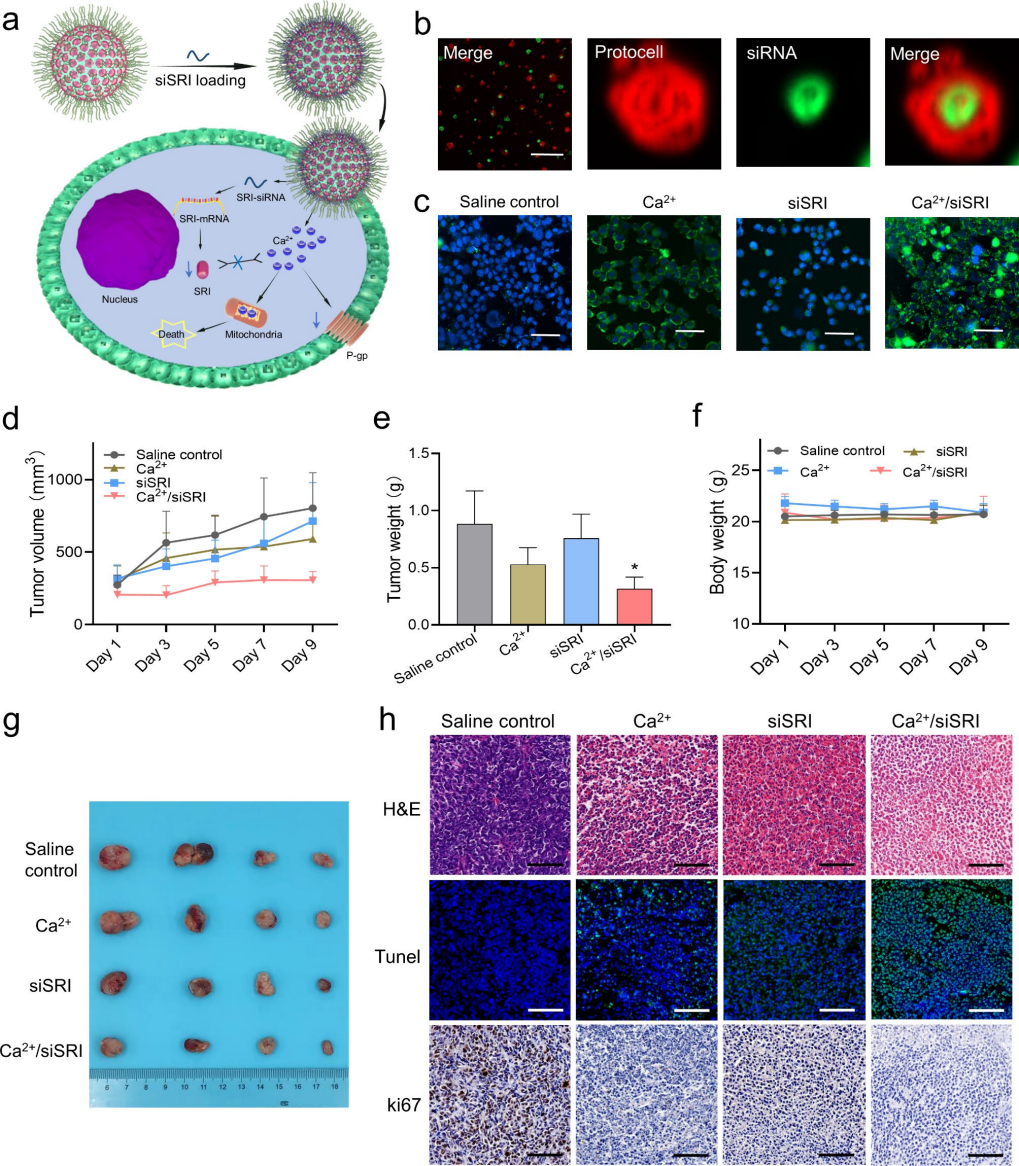
Fabricating synthetic protocells by NISA method, and restoring calcium homeostasis in chemoresistant cancer cells. **(a)** Schematic illustration of synthetic protocells restoring calcium homeostasis and overcoming drug resistance. **(b)** Confocal images of fam-siRNA loaded synthetic protocells. Scale bar, 100 μm. **(c)** Confocal images reflect the calcium ion concentration in various groups. Scale bar, 100 μm. **(d)** Tumor volume of various groups during treatment. **(e)** Tumor weight from various groups after treatment. **(f)** Body weight from various groups during treatment. **(g)** Pictures of tumors harvested from the treated mice after treatment. **(h)** H&E, Tunnel, and Ki67 stained A549/PTX tumor slices from various groups. Scale bar, 100 μm

The protocell has the natural advantage as a drug carrier because of its saccular structure and imitative membrane [37–39], chemotherapeutic drugs such as doxorubicin can be easily loaded into protocells [40]. An imbalance of calcium homeostasis caused by reduced calcium ion concentration is common in drug-resistant cells [41]. In order to supplement calcium ions to reverse drug resistance, we loaded siRNA of calcium-binding protein (SRI) in protocell to knock down SRI. The schematic is shown in Fig. 5a, as the matrigel making up protocells is mainly extracellular matrix proteins, which can be degraded by proteolytic enzymes such as matrix metalloproteinases in vivo, leading to the breakdown of the protocells. After the protocells were degraded, siSRI was released. Decreased SRI can release more calcium ions, and the chelated calcium ions in protocells can also increase calcium ions in drug-resistant cancer cells. Laser confocal observation showed that fam-labeled siRNA appeared in the core of the protocell (Fig. 5b), this phenomenon indicates that the combination of siRNA and cationic lipid DOTAP occurs inside the protocell, because the gel microsphere is negatively charged due to the large number of carboxyl groups of chelating agent DTPA, so the cationic lipid DOTAP is more inclined to appear inside the membrane of the protocell compared with the neutral lipid DOPE. To determine whether the protocells could increase calcium ion concentration in drug-resistant tumor cells, the protocell was incubated with drug-resistant tumor cells, and a calcium fluorescence probe was utilized to detect the intracellular calcium ion concentration. Paclitaxel-resistant A549 cell line (A549/PTX) was selected for the in vivo measurement of protocells. A549/PTX cells have a fast growth rate and a short tumor-forming period. The intra-tumor injection was carried out when the tumor volume reached 80–100 mm3. In order to inhibit the rapid growth of the tumor and avoid the tumor size in the saline control group exceeding the capacity of the mice, the injection was designed to be performed 5 times once every 2 days, and the mice were harvested after 11 days of treatment. The results showed that the Ca^2+^/siSRI group had the highest intracellular calcium ion concentration compared to other groups (Fig. 5c). Through in vivo experiments, we found that the Ca^2+^ group and especially Ca^2+^/siSRI group significantly inhibited the growth of drug-resistant tumors, in the case of no significant difference in mouse body weight of different groups (Fig. 5d g, Fig. S6). Tunel staining showed the most obvious apoptosis in tumor tissue of Ca^2+^/siSRI group, while ki67 showed the slowest growth rate in Ca^2+^/siSRI group (Fig. 5h). The detection of heart, liver, spleen, lung, and kidney showed no obvious damage (Fig. S7), which proved the safety of siRNA loaded protocell. All the results indicated that the synthetic protocells upon the NISA method could be used as a gene or drug carrier for the effective treatment of refractory diseases.

## Discussion

The preparation strategies of protocells include top-down and bottom-up methods. In this study, we developed non-interfacial self-assembly (NISA) of microdroplets as a step toward synthetic protocells, which consists of lipid molecules on its surface and hydrogels inside. The NISA method is suitable for both top-down and bottom-up methods to build protocells, and compared with the traditional methods such as the hydration method, phase transfer method, microfluidic method, colloid and condensate [5], the NISA method fabricates protocells with more similar morphology to the native cells. In addition to the structure, synthetic protocell exhibited similar functions to living cells, which make it have potential applications in immunotherapy, cell therapy, gene therapy, etc.

The NISA-based synthetic protocells can synthesize mRNA by using the encapsulated DNA templates and RNA enzymes. mRNA vaccine is an important vaccine technology, because of its safety, convenience, and high efficiency [42–44]. However, exposed mRNA is easily degraded by extracellular ribonuclease during the loading and administration process [45, 46]. mRNA stability has been a stumbling block to its development. The use of mRNA carrier is an important way to solve the stability problem [47, 48], such as lipid nanoparticles [49], which was widely used for the delivery of mRNA vaccines, however, common mRNA carriers carry a limited amount of mRNA [50]. Therefore, a protocell-based mRNA bomb was reported herein which was prepared based on the NISA method. The NISA-based technique achieved mRNA transcription and loading in synthetic protocells, which avoids the possibility of mRNA degradation during the loading and transporting process. Since the nucleotides outside the synthetic protocells can continuously pass through the semi-permeable membrane to participate in the synthesis of mRNA, this method can achieve the maximum theoretical load efficiency of mRNA. In clinical practice, mRNA encoding specific antigens such as viruses, bacteria, or even tumor cells can be loaded into synthetic protocells using the NISA method, as a potential mRNA vaccine, this NISA-based synthetic protocells is expected to be used in non-viral immunotherapy.

In addition, the NISA-based synthetic protocells technique can be used to refabricate cancer cells. The technique involves extracting the cancer cell’s membrane and entire genes, then mixing the DNA with a hydrogel and wrapping the membrane around the gel’s surface to reorganize it into a protocell. The refabricate cells is greatly retained the immunogenicity of cancer antigen, realizing the purpose of killing cancer cells by enhancing the patient’s immune system. In addition, immune adjuvants, drugs, DNA, and RNA can also be loaded into the synthetic protocells to enhance immune function and achieve better therapeutic effects. Furthermore, the patient’s own antigens are used for immunotherapy that achieve the purpose of safe and accurate treatment. In clinical practice, cancer patients’ tumor cell surface antigens can be extracted, and construct synthetic protocells using the NISA method. The protocells can then be injected into patients to activate the patient’s immune system and improve its ability to attack cancer cells. Therefore, as a potential cancer vaccine, this NISA-based synthetic protocell is expected to be used in clinical cancer immunotherapy.

Furthermore, NISA-based synthetic protocells can load genes or drugs to treat difficult-to-cure tumors. The decrease of calcium ion concentration in the cytoplasm and the increase of calcium-binding protein expression lead to calcium ion imbalance, which is an important cause of tumor drug resistance. By loading siRNA-SRI to knock down the calcium-binding protein, and utilizing its chelated calcium ions to increase the calcium ion concentration in drug-resistant cells, the synthetic protocells can restore calcium homeostasis of drug-resistant cells and reverse its drug resistance. This approach avoids the use of chemotherapy drugs with high side effects and achieves effective reversal of tumor drug resistance. Therefore, NISA-based synthetic protocells envelopes the drugs and releases them in the tumor, which improve the efficiency of drugs and reduce side effects. Thus, these NISA-based synthetic protocells are expected to be used in the clinical treatment of drug-resistant tumors.

In sum, compared with traditional technology, the NISA-based technique achieved synchronous transcription and loading of mRNA in synthetic protocells, avoiding the degradation of mRNA during loading and transportation. Besides, artificial cancer cell fabricated via the NISA method has a similar structure as that of the native cancer cell, that greatly retain the immunogenicity of cancer antigens, which is more conducive to achieve a better immunotherapy effect. Moreover, NISA-based synthetic protocells can load genes and drugs to achieve the purpose of combined treatment of cancers.

## Conclusion

Herein, we developed non-interfacial self-assembly (NISA) of microdroplets as a step toward synthetic protocells and explored a series of biological applications. Firstly, the synthetic protocells were utilized to transcribe and load mRNA, as a potential mRNA vaccine, the NISA-based synthetic protocell is expected to be used in non-viral immunotherapy. Secondly, the synthetic protocells technique can be used to refabricate cancer cells, this NISA-based synthetic protocell can work as a personalized cancer vaccine for clinical cancer immunotherapy. Thirdly, the synthetic protocell can deliver genes and drugs to treat difficult-to-cure tumors, as a drug carrier, these NISA-based synthetic protocells are expected to be used in the clinical treatment of drug-resistant tumors. In conclusion, NISA-based synthetic protocell technology is a potential clinical therapeutic approach, which can be used in immunotherapy, cell therapy, chemotherapy, gene therapy, etc.

## Materials and methods

### Fabrication of synthetic protocells upon NISA method

A mixture of 800 mg of DSPE-PEG-DTPA (Biological Technology, Xian, China) and 400 µL of Cremophor EL (Innochem, Beijing, China) was dissolved in 1 mL of ethanol, the mixture was then vibrated until it is completely dispersed at 60 ℃. After cooling to 4 ℃, the mixture along with 1.5 mL of matrigel (Becton, Dickinson and Company, USA) was transferred to 6 mL of calcium chloride solution (60 mg/ mL in normal saline). Then the mixture was transferred to 40 mL of PFOB (Aladdin, Shanghai, China) and shaken at room temperature for 30 min until it became a uniform and stable emulsion. The PFOB solution was then transferred to an aqueous solution containing 10 mg/mL of DOPE (A.V.T. (Shanghai) Pharmaceutical Co., Ltd., Shanghai, China) and 10 mg/mL of DOTAP (A.V.T. (Shanghai) Pharmaceutical Co., Ltd., Shanghai, China). Finally, the synthetic protocells were obtained in an aqueous solution after removing the PFOB.

### Transcription and expression of synthetic protocells

The synthetic protocells were fabricated as above, the difference is that 200 ng of DNA template and RNA transcriptase were added into the gel mixture during the preparation progress. After protocells preparation, nucleotides were added into the protocells solution and then incubated at 37 ℃ for 4 h, mRNA was transcribed inside the protocells.

### Fabricated artificial cancer cells by the NISA method

After the cells were lysed by sodium dodecyl sulfate (SDS) lysis buffer, cell membranes were collected by high-speed centrifugation. DNA was extracted using a DNA extraction Kit (Qiagen, Germany) according to the manufacturer’s instructions. DNA was added to the mixed gel, and the lipid molecules in the water were replaced by cell membranes to prepare artificial cells using the same method as above.

### Fabrication of siRNA-SRI loaded synthetic protocells upon NISA method

The synthetic protocells were fabricated as above, 5 µL of synthetic protocells (80 mg/mL) and 5 µL of siRNA-SRI (20 µM) were mixed to form the siRNA-SRI loaded synthetic protocells (Ca^2+^/siSRI group). siRNA-SRI loaded alone synthetic protocell (siSRI group) was obtained by simply removing calcium chloride during the preparation process. The original synthetic protocell was labeled as a Ca^2+^ group.

### Characterization of synthetic protocells upon the NISA method

Optical and fluorescence microscopy were recorded with a laser scanning confocal microscope (SP8 STED 3X, Biotek, GER). The diameter of the synthetic protocells was analyzed using ImageJ software, synthetic protocells were dissolved in pure water at a concentration of 1 mg/mL at 25 °C. The morphology of the synthetic protocells was characterized by using an atomic force microscope (MFP-3D, OXFORD, USA). Flow cytometry/fluorescence-activated cell sorting (FACS) was measured via flow cytometry (Gallios, Beckman Coulter, Inc., Brea, CA, USA).

### PCR amplification and agarose gel retardation assay

PCR amplification was performed using PCR Amplifier (ExCell Bio, China). The PCR primer sequences are shown in Supplementary Table S1. PCR products were loaded onto a 1.2% agarose gel for gel retardation assay. The protocells were digested by trypsin and mRNA was purified according to the instructions of the in vitro transcription kit. One µL of mRNA was loaded onto a 1.2% agarose gel, and electrophoretic separation was performed at 140 V for 20 min. The gel was then detected and photographed using GelDoc EZ Transmissometer (Bio-Rad, USA).

### RNA extraction and quantitative real-time PCR

RNA extraction was performed using an RNA-Quick Purification Kit (ES Science, Shanghai, China), and cDNA was synthesized using a PrimeScript Reverse Transcriptase Kit (TaKaRa, Japan). Real-time PCR was performed using a qPCR RT kit (Mei5 Biotechnology, Beijing, China) according to the manufacturer’s instructions, and the gene expression was evaluated using a Real Time PCR System (7300, Applied Biosystems, CA, USA). The PCR primer sequences are shown in Supplementary Table S1.

### Protein extraction and Western blot analysis

After the cells were lysed by sodium dodecyl sulfate (SDS) lysis buffer, the lysate was performed by ultrasonic and centrifuge. Protein in the lysate was then separated by SDS-polyacrylamide gel electrophoresis. The primary antibodies were rabbit anti-CD44 (1:5000 dilution, CST, USA) and mouse anti-β-actin (1:5000 dilution, CST, USA) which was used as an internal reference. The secondary antibodies were HRP-conjugated goat anti-mouse IgG (1:5,000 dilution, Proteintech, USA) and HRP-conjugated goat anti-rabbit IgG (1:5,000 dilution, Proteintech, USA). Protein bands were colored using Immobilon™ HRP Substrate (Millipore, MASS, USA), and photographed using Tanon-4500 Gel Imaging System (Tanon Science & Technology, Shanghai, China).

### Calcium ion analysis

The cells were seeded into 6-well plates at a density of 2 × 10^5^ cells /well. After incubation for 24 h, the cells are stained with a calcium concentration detection kit (Beyotime Biotechnology, Shanghai, China). After washing three times with cold PBS, the cells were photographed using a laser scanning confocal microscope.

### In vivo therapy and pathological assessment

Animal ethics are approved by the Ethics Committee of Shanghai Public Health Clinical Center. BALB/c nude mice were purchased from Shanghai B&K Laboratory Animal Co., LTD. (Shanghai, China). 100 µL of PBS solution containing approximately 3 × 10^6^ of A549/PTX cells was injected into the right side of each mouse. Three weeks later, the tumor-bearing mice were randomly divided into 4 groups, including a saline control group, Ca^2+^ group, siSRI group, and Ca^2+^/siSRI group. The sequence of siSRI is shown in Supplementary Table S2. Mice were injected with 100 µL of saline, Ca^2+^, siSRI, or Ca^2+^/siSRI loaded synthetic protocells (80 mg/mL) for each time. The intratumor administration was performed 5 times once every 2 days. Tumor volume was monitored every 2 days, and the tumor volume was calculated by the formula V = ab^2^/2, where V refers to the volume, and a and b are the tumor length and tumor width, respectively. The mice were killed after treatment, and the tumors were harvested and photographed.

Tumor tissue was performed pathological examination at Ruibu Biological Technology Co., LTD (Shanghai, China). The tumor tissue was firstly fixed with formalin and embedded in paraffin, then the tumor tissue specimens were stained with H&E and Ki67, and photographed under a microscope.

### Statistical analysis

Particle size, tumor volume, tumor weight, and body weight were presented as the mean ± standard deviation (SD), and the qRT-PCR was presented as mean ± standard error (SE). Student’s *t-*test was used for statistical analysis of differences between groups, and p < 0.05 was considered statistically significant. Flow cytometry data were analyzed using FlowJo, and graphs were drawn using GraphPad and Origin software.

### Electronic supplementary material

Below is the link to the electronic supplementary material.

Additional file 1: table S1 PCR Primer sequences. Additional file 1: table S2 siRNA sequence. Additional file 1: figure S1 The chemical structure of DSPE-PEG-DTPA. Additional file 1: figure S2 The hydrogel was transferred into perfluorooctyl bromide (PFOB) at the beginning (a), and after shaking at room temperature for 30 min (b). Additional file 1: figure S3 Laser confocal micrograph of A2780 and A2780/PTX, CD44 protein was highly expressed in A2780/PTX analyzed by immunofluorescence. Additional file 1: figure S4 Laser confocal micrograph of protocell A2780 and protocell A2780/PTX and the expression of CD44 protein was analyzed by immunofluorescence. Additional file 1: figure S5Microscopic photograph of artificial cancer cells (protocell A2780) stored for 2 weeks (a) and 3 months (b) at 4 ℃ Additional file 1: figure S6 Picture of the A549/PTX tumor-bearing mice after treatment. Additional file 1: figure S7 H&E stained heart, liver, spleen, lung, and kidney tumor specimens harvested from the treated mice.

Additional file 2: video S1 Confocal micrograph of synthetic protocells.

Additional file 3: video S2 Confocal micrograph of synthetic protocells after drying.

Additional file 4: video S3 Confocal micrograph of synthetic protocell post-transcription.

Additional file 5: video S4 Dynamic changes of 293T cells incubating with synthetic protocells in 4 h.

Additional file 6: video S5 Confocal micrograph of A2780 and A2780/PTX.

Additional file 7: video S6 Confocal micrograph of protocell A2780 and protocell A2780/PTX.

## Data Availability

The datasets during and/or analyzed during the current study are available from the corresponding author upon reasonable request.
