# Correction: Non-interfacial self-assembly of synthetic protocells

**DOI:** 10.1186/s40824-023-00436-0

**Published:** 2023-10-02

**Authors:** Xiaolin Xu, Wencai Guan, Xiaolei Yu, Guoxiong Xu, Chenglong Wang

**Affiliations:** 1 Research Center for Clinical Medicine, Jinshan Hospital, Fudan University, Shanghai, 201508 P.R. China; 2 The State Key Laboratory of Metal Matrix Composites, School of Materials Science and Engineering, Shanghai Jiao Tong University, Shanghai, 200240 P. R. China

**Biomaterials Research (2023) 27:64**

10.1186/s40824-023-00402-w

The authors regret to report the grant number “21871180” in the original article [1] should instead state “81872121”.

