# Supplementary material for: Non-interfacial self-assembly of synthetic protocells
Source: Biomater Res. 2023 Jul 3;27:64. doi: 10.1186/s40824-023-00402-w (PMC10318706; doi:10.1186/s40824-023-00402-w)
Supplement: Supplementary file 7 — Additional file 7: video S6 Confocal micrograph of protocell A2780 and protocell A2780/PTX. [file 40824_2023_402_MOESM7_ESM.docx]

**Non-interfacial self-assembly of** **synthetic protocells**

Xiaolin Xu^1^, Wencai Guan^1^, Xiaolei Yu^2^, Guoxiong Xu^1, *^, Chenglong Wang^1, *^

^1^ Research Center for Clinical Medicine, Jinshan Hospital, Fudan University, Shanghai 201508, P.R. China.

^2^ The State Key Laboratory of Metal Matrix Composites, School of Materials Science and Engineering, Shanghai Jiao Tong University, Shanghai 200240, P. R. China.

* Corresponding authors.

*E-mail addresses*: guoxiong.xu@fudan.edu.cn (G. Xu), aaron-wang@alumni.sjtu.edu.cn (C. Wang).

**Table S1.** PCR Primer sequences

| Name | Primer Direction | Sequence  (5’ → 3’) | GenBank Accession # |
| --- | --- | --- | --- |
| GFP fragment | Forward | gaccatgattacgccAATACGACTCACTATAGGggtttagtgaaccgtcagatcc | / |
|  | Reverse | aaaacgacggccagtGAATTCcgccttaagatacattgatgagtttgg |  |
| CD44 | Forward | CTGCCGCTTTGCAGGTGTA | NM_001001392 |
|  | Reverse | CATTGTGGGCAAGGTGCTATT |  |
| Sorcin | Forward | GGACAAACTCAGGATCCGCTGTA | NM_003130.4 |
|  | Reverse | GCCGGCAAGTCTCCAGGTTA |  |
| GAPDH | Forward | GCACCGTCAAGGCTGAGAAC | NM_002046.7 |
|  | Reverse | TGGTGAAGACGCCAGTGGA |  |

**Table S2.** siRNA sequence

| Name | Sequence (5’ → 3’) | Target position |
| --- | --- | --- |
| Sorcin |  |  |
| Sense-1 | GCUGGAGACAACACUUUAUTT | nt 323-341 |
| Antisense-1 | AUAAAGUAUUAUCUCCAGCTT |  |

**Figure S1.** The chemical structure of DSPE-PEG-DTPA.


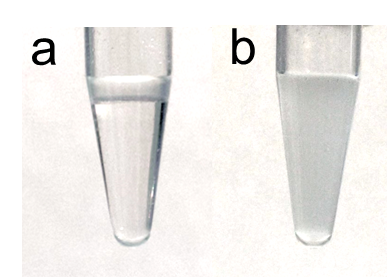


**Figure S2.** The hydrogel was transferred into perfluorooctyl bromide (PFOB) at the beginning (**a**), and after shaking at room temperature for 30 min (**b**).


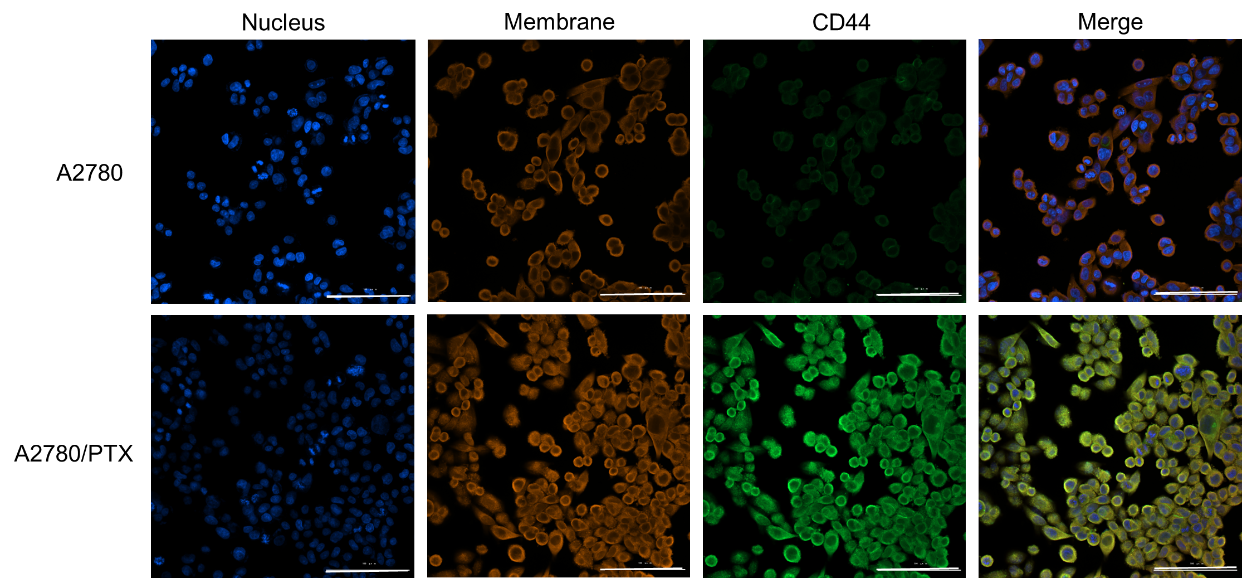


**Figure S3.** Laser confocal micrograph of A2780 and A2780/PTX, CD44 protein was highly expressed in A2780/PTX analyzed by immunofluorescence. Scale bar, 100 μm.


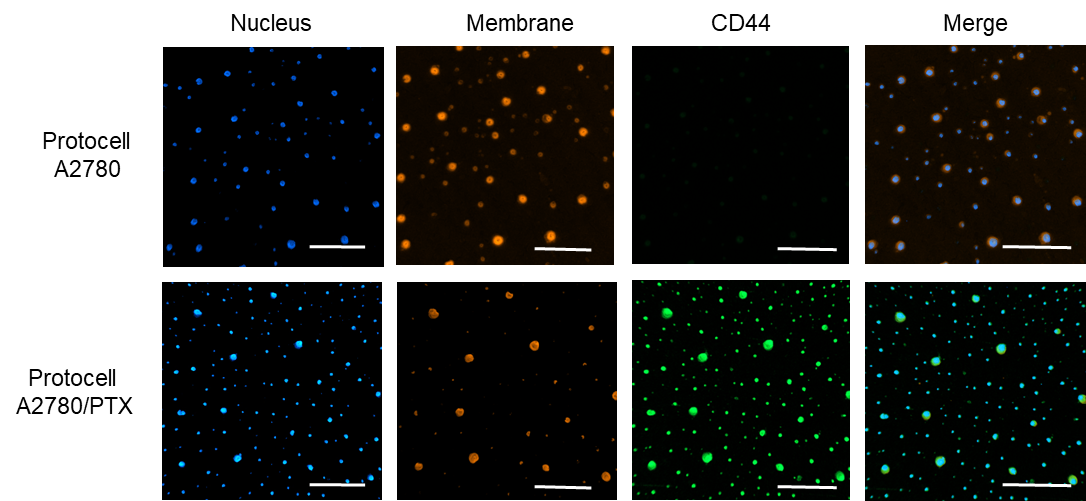


**Figure S4.** Laser confocal micrographs of protocell A2780 and protocell A2780/PTX. The expression of CD44 protein was analyzed by immunofluorescence. Scale bar, 100 μm.


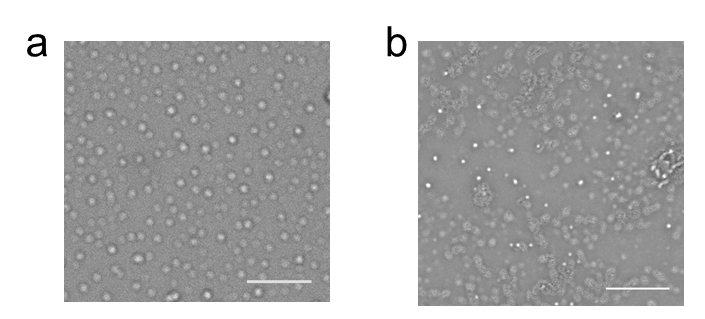


**Figure S5.** Microscopic photograph of artificial cancer cells (protocell A2780) stored for 2 weeks (a) and 3 months (b) at 4 ℃.


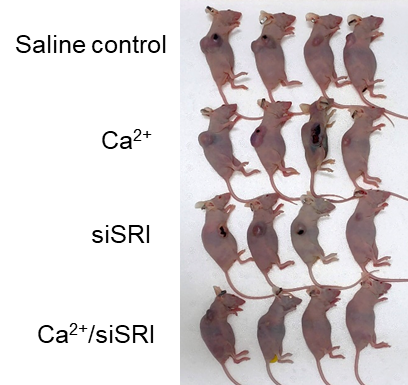


**Figure S6.** Picture of the A549/PTX tumor-bearing mice after treatment.

**
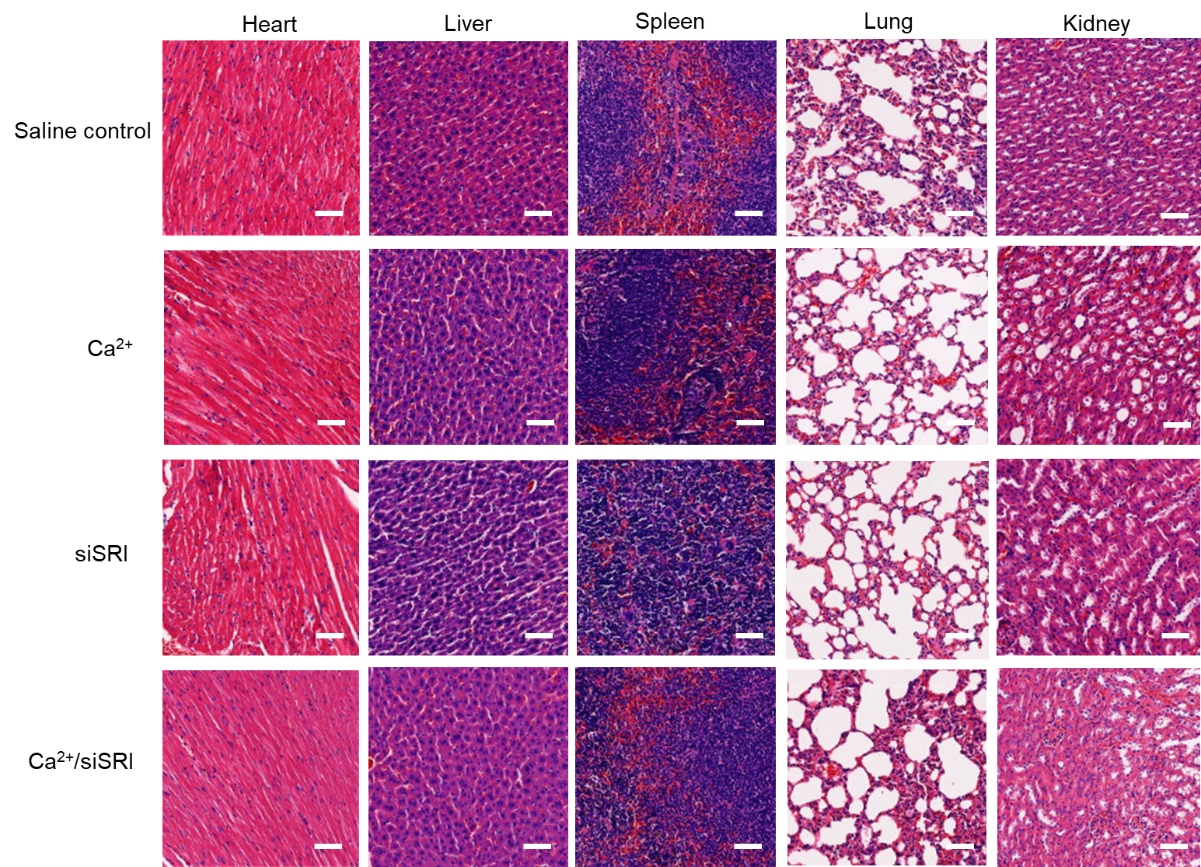
**

**Figure S7.** H&E stained heart, liver, spleen, lung, and kidney tumor specimens harvested from the treated mice. Scale bar, 50 μm.
